# Genesee County Medical Society

**Published:** 1872-01

**Authors:** 


### Genesee Co. Medical Society.

PAVILION, N. Y., Jan. 15, 1872.

DEAR DOCTOR:—Will you please publish the following, which I am authorized to send you?

Yours truly,

J. F. MINER. W. B. SPRAGUE.

At the regular semi-annual meeting held at the St. James Hotel, in the village of Batavia, on Tuesday, Jan. 9th, 1872, the following preamble and resolution was offered by Dr. O. R. Croff, and unanimously adopted:

*Whereas,* At a special meeting of the Genesee County Medical Society, held July 5, 1870, a certain motion rescinding the resolution of expulsion in reference to Dr. N. G. Clark was passed, and whereas the minutes were not ordered published as should have been done in justice to Dr. Clark and to the Society:

*Resolved,* That the fact of such rescinding of a motion which was illegally made for expulsion of Dr. Clark be published in the *Buffalo Medical Journal,* and that the Society regret that the publication of such action was unintentionally deferred.

Meeting then adjourned to the second Tuesday of February next.

WM. B. SPRAGUE, Chairman.

L. L. TOZIER, Secretary.

